# Possibility of Isolated Mung Bean Protein as a Main Raw Material in the Production of an Extruded High-Moisture Meat Analog

**DOI:** 10.3390/foods13142167

**Published:** 2024-07-09

**Authors:** Nam-Ki Hwang, Bon-Jae Gu, Yu Zhang, Gi-Hyung Ryu

**Affiliations:** Department of Food Science and Technology, Food and Feed Extrusion Research Center, Kongju National University, Yesan 32439, Republic of Koreabon-jae.gu@kongju.ac.kr (B.-J.G.);

**Keywords:** high-moisture extrusion cooking, meat analog, isolated mung bean protein, isolated soy protein, physicochemical properties

## Abstract

As consumer demand for meat analogs continues to grow, various plant proteins are being explored for their production. This study uses isolated mung bean protein (IMBP) to replace isolated soy protein (ISP), investigating the effects of IMBP content (0%, 10%, 20%, 30%, 40%, and 50%) on the physicochemical and textural properties of high-moisture meat analogs (HMMAs) and exploring the potential of IMBP in the development and production of meat analogs. The results show that IMBP can bind water and cause protein denaturation, thus requiring more time and higher temperatures to be formed compared to HMMAs without IMBP. Additionally, increasing the IMBP content improves the gelling ability, thereby increasing the input of specific mechanical energy. As the IMBP content increases, the fibrous structure of the HMMA also increases. When the IMBP content reaches 40–50%, the most meat-like fibrous structure is observed. The water-holding capacity, water absorption capacity, springiness, and cohesiveness are negatively correlated with the IMBP content, while the oil absorption capacity is positively correlated with it. The integrity index and nitrogen solubility index show opposite trends with the increase in the IMBP content. When the IMBP content is 50%, the springiness and chewiness are the lowest, and the cutting strength is also the lowest, but the sample has a rich fibrous content, indicating that the HMMA with 50% IMBP content is soft and juicy. In conclusion, IMBP has the potential to be a substitute for ISP in the production of HMMAs.

## 1. Introduction

According to the United Nations’ [1] projections, the global population is expected to reach 10 billion by 2050, with the global demand for meat reaching approximately 455 million tons. This represents a 76% increase compared to 2005 [2]. To meet the continuously growing demand for meat, it is necessary to increase livestock production by 50–73% [3]. However, livestock production emits three major greenhouse gases: carbon dioxide (CO_2_), methane (CH_4_), and nitrous oxide (N_2_O), leading to environmental issues [4,5]. Therefore, considering the disparity between future meat demand and current meat supply capabilities, there is an increasing need to consider meat analogs as a new source of protein [6].

Meat analogs are plant-based foods that mimic the appearance, flavor, and texture of meat [7]. Extrusion cooking is one of the most used manufacturing processes for producing meat analogs, which can be classified into low-moisture extrusion cooking (LMEC) and high-moisture extrusion cooking (HMEC) based on different moisture contents. Moisture is considered a crucial factor in the extrusion process. Generally, the moisture content is in the range of 10–30% for LMEC and in the range of 50–70% for HMEC. It has been reported that LMEC using short dies may result in expansion [8], while HMEC using long cooling dies can generate meat-like fibrous structures without expansion [9].

Soybeans [10], peas [11], wheat gluten [12], and peanut protein are major plant-based proteins utilized in the production of contemporary meat analogs [13]. Isolated soy protein (ISP) has garnered attention due to its high protein content exceeding 90%, achieved through the removal of oligosaccharides and water-soluble polysaccharides [14]. Moreover, its inherent attributes, such as isoflavones and saponins, contribute to its antioxidative and immunomodulatory functionalities [15]. Nevertheless, concerns regarding the distinctive flavor and bitterness of soybeans [16], as well as issues related to allergies and potential genetically modified organisms, have spurred interest in alternative plant proteins, including mung beans [17].

Mung beans, being a staple crop widely cultivated and consumed in most Asian countries, hold undeniable significance [18]. Studies indicate that mung beans possess various health benefits, including lowering blood sugar levels, inhibiting melanin production, immunomodulation, and hepatoprotection [19]. Isolated mung bean protein (IMBP) is recognized as a high-quality protein, abundant in essential amino acids such as proline, glutamic acid, arginine, leucine, and phenylalanine [20]. Furthermore, it has been reported that the amino acid composition of mung bean protein is like that of soybean protein [21]. The primary component of mung bean protein, constituting over 80%, is 8S globulin. The sequence similarity of the 8S globulin to β-conglycinin, a major protein in soybeans, is 68%, with a structural similarity of 68% as well. The potential health benefits of mung bean protein are estimated to be four times that of β-conglycinin, which accounts for 20% of total soybean protein [22]. The research conducted by Brishti et al. [23] successfully demonstrated the feasibility of optimizing the production of texturized mung bean protein (TMBP) from mung bean protein. TMBP can serve as a meat thickener and is considered a healthier alternative to animal protein. Additionally, Brishti et al. [23] reported that extrusion at 49.3% moisture content can result in the production of TMBP with desirable characteristics, including partial denaturation, the formation of small aggregates, enhanced solubility, and digestibility. TMBP also exhibits strong gel-forming behavior.

However, there is currently a lack of research on high-moisture meat analogs based on isolated mung bean protein content. Therefore, this study aims to investigate the physicochemical properties of extruded high-moisture meat analogs based on varying levels of isolated mung bean content.

## 2. Materials and Methods

### 2.1. Materials

The raw material ratio of isolated mung bean protein (IMBP)/ isolated soybean protein (ISP): wheat gluten (WG): corn starch (CS) was 50: 40: 10. ISP (Pingdingshan Tianjing Plant Albumen Co., Ltd., Pingdingshan, China) was replaced with IMBP (Harbin Hada Starch Co., Ltd., Harbin, China) at inclusion levels of 0%, 10%, 20%, 30%, 40%, and 50%, while WG (Roquette Freres, Lestrem, France) and CS (Samyang Ltd., Ulsan, Republic of Korea) were fixed and mixed in a ratio of 40:10. The dry basis crude protein content, ash content, and crude fat content of IMBP are 80%, 7%, and 2%, respectively, while those of ISP are 90%, 5%, and 1%, respectively.

### 2.2. High-Moisture Extrusion Process

**Figure 1:**
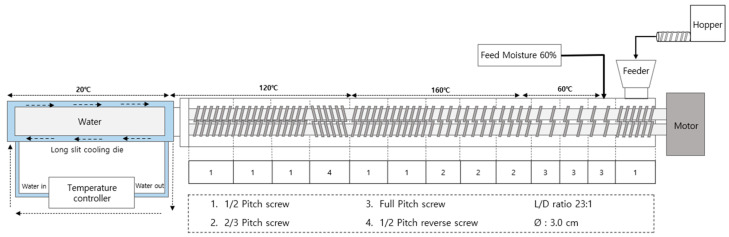
Schematic diagram of the high-moisture extrusion system with details of the cooling die and screw configuration.

The experiment was conducted using a co-rotating twin-screw extruder (THK31T-No.5, Incheon Machinery Co., Incheon, Republic of Korea), with a screw length-to-diameter ratio of 23:1 and a screw diameter of 3 cm. The cooling die and screw configuration of extruder are illustrated in Figure 1. The extrusion parameters included: a feed rate of 100 g/min, feed moisture content of 60%, and screw speed of 150 rpm. The cooling die temperature was maintained at 20 °C, utilizing a water circulator (Duksan Cotran Co., Daegu, Republic of Korea). Following extrusion, the HMMA samples were cut into 1 × 1 × 1 cm blocks, with a portion stored under sealed conditions at 4 °C for integrity index and texture profile analysis (TPA) measurements, while another portion was freeze-dried for water holding capacity (WHC) assessment. Subsequently, a portion of the freeze-dried HMMAs was ground using a grinder (FM-909T, Hanil Electric Co., Wonju, Republic of Korea), sieved, and samples with a particle size of 50–70 mesh were utilized for measuring the water absorption capacity (WAC), oil absorption capacity (OAC), and nitrogen solubility index (NSI).

### 2.3. Viscosity

The viscosities of ISP, IMBP, and their mixtures (IMBP 0%, 30%, and 50%) were measured using a Rapid Visco Analyzer (TecMaster, Perten Instruments, Perkin Elmer, NSW, Australia). The measurement protocol was adapted from the method of Liu et al. [24], with slight modifications. A total of 3 g of samples were uniformly mixed with 25 mL of distilled water and stirred at a speed of 160 rpm for 13 min. Heating and cooling conditions involved maintaining the initial temperature at 50 °C for 1 min, followed by gradual increase to 95 °C over 4 min and then holding for 3 min. Subsequently, the temperature was reduced to the initial temperature of 50 °C over 4 min and maintained for an additional 2 min. The analysis of the RVA curve data was conducted to measure the peak viscosity, final viscosity, and setback.

### 2.4. Specific Mechanical Energy

The specific mechanical energy (SME), defined as the electrical energy consumed per unit mass during the extrusion process, was measured following the methodology proposed by Ryu and Mulvaney [25]. The SME is represented by Equation (1).

SME input (kJ/kg) = (E − E_0_)/P_R_, (1)

where E is the electric power when input to the material (kJ/s), E_0_ is the electric power when idle (kJ/s), and P_R_ is the production rate (kg/s).

### 2.5. Water Holding Capacity

To assess the WHC of the HMMAs, modifications were made to the method proposed by [9]. A total of 3 g of freeze-dried block-shaped HMMAs (on a dry basis) were mixed with 100 mL of distilled water. The mixture was hydrated at 50 °C using a water bath (SHWB-45, Lab House Co., Ltd., Pocheon, Republic of Korea) for 16 h. Subsequently, the sample was filtered through a 20-mesh sieve for 15 min to remove excess moisture. The WHC is expressed by Equation (2). Three replicate measurements were conducted, and the average value was calculated.

WHC (g/g) = (Wet sample wt. − Dry sample wt.)/Dry sample wt.(2)

### 2.6. Texture Profile Analysis and Cutting Strength

The HMMA texture was assessed using a rheometer (Compac-100Ⅱ, Sun Sci Corporation, Tokyo, Japan). A probe with a diameter of 2.5 cm was utilized to measure the springiness, cohesiveness, and chewiness of the samples. The measurements were conducted at a maximum stress of 10 kg using the probe. Additionally, the cutting strength in both the vertical and parallel directions were measured using a probe with the dimensions of 7.5 mm × 38.3 mm at a maximum stress of 2 kg. Subsequently, the springiness, cohesiveness, chewiness, and cutting strength were calculated using Equation (3), Equation (4), Equation (5), and Equation (6), respectively. Ten measurements were conducted for each sample, and after excluding the maximum and minimum values, the average was derived from the remaining six data points.

Springiness (%) = D_2_/D_1_ × 100, (3)

where D_1_ is the distance of the first-occurred maximum stress and D_2_ is the distance of the second-occurred maximum stress.

Cohesiveness (%) = A_2_/A_1_ × 100, (4)

where A_1_ is the area of the first-occurred maximum stress and A_2_ is the area of the second-occurred maximum stress.

Chewiness (g) = Springiness × Cohesiveness × Maximum stress/10000,(5)

Cutting strength (g/cm^2^) = Maximum stress/Cross-sectional area. (6)

### 2.7. Water Absorption Capacity and Oil Absorption Capacity

The water absorption capacity (WAC) and oil absorption capacity (OAC) were measured according to the modified method by Samard and Ryu [26]. A total of 0.5 g of ground samples were mixed with 5 mL of distilled water using a vortex mixer (SI-0246A, Vortex-Genie-2, Scientific Industries Inc., Bohemia, NY, USA) for 1 min. Subsequently, the mixture was vibrated on a shaker (SI-300R, Jelotech, Gangneung, Republic of Korea) at 30 °C for 30 min, followed by centrifugation at 3000 rpm for 30 min and discarding of the supernatant. Simultaneously, the same procedure was repeated using soybean oil instead of distilled water. The WAC and OAC of the sediment were calculated using Equations (7) and Equation (8), respectively. The density of water used in the experiment was 1 g/cm^3^, and the density of soybean oil was 0.90 g/cm^3^. The experiment was repeated three times, and the average value was calculated.

WAC (g/g) = Weight of sediment/Weight of dry solid,(7)

OAC (g/g) = Weight of sediment/Weight of dry solid.(8)

### 2.8. Integrity Index

The integrity index was measured following the method by Gu and Ryu [27]. It evaluates the resistance of the HMMA’s fibrous structure to high temperature and pressure, as well as its homogeneity. A total of 4 g of freeze-dried block-shaped sample (on a dry basis) were placed in 100 mL of distilled water and heated under high pressure at 121 °C for 15 min using an autoclave. After cooling the samples in running water for 30 s, they were homogenized in a beaker containing 100 mL of distilled water using a homogenizer (IKA-T10B, IKA Co., Staufen, Germany) and filtered through a 20-mesh sieve. The residue was rinsed once with running water for 30 s and then dried at 105 °C for 10 h. The experiment was repeated three times, and the average value was calculated.

Integrity index (%) = Dry residue wt./Sample wt. × 100.(9)

### 2.9. Nitrogen Solubility Index

The nitrogen solubility index (NSI) was determined using the modified method by Căpriță, and Crețescu [28]. To measure the content of soluble nitrogen, 0.1 g of powdered samples were mixed with 5 mL of 0.5% KOH solution and stirred at 120 rpm for 20 min using a shaker (SI-300R, Jelotech, Gangneung, Republic of Korea) at 30 °C. The mixture was then centrifuged at 3000 rpm for 30 min. Subsequently, 0.05 mL of supernatant were collected, and the content of soluble nitrogen was determined using the anthrone method according to Starcher [29].

The method for determining the total nitrogen content involved completely hydrolyzing 0.1 g of powdered sample in 6 N hydrochloric acid (250 rpm, 100 °C, 24 h). After adding 5 mL of distilled water, the mixture was centrifuged at 3000 rpm for 30 min, and 0.05 mL of supernatant were collected. The total nitrogen content was then determined using the anthrone method. The calculation formula for NSI is as follows in Equation (10).

NSI (%) = Soluble nitrogen content/Total nitrogen content × 100.(10)

### 2.10. Statistical Analysis

The statistical analysis of the results was conducted using the SPSS software (version 27.0, IBM-SPSS, Somers, New York, NY, USA), which is a statistical package commonly used in the social sciences. A one-way analysis of variance (ANOVA) was performed, followed by Duncan’s multiple range test to assess significant differences among groups, with a significance level set at *p* < 0.05.

## 3. Results

### 3.1. Viscosity

**Figure 2:**
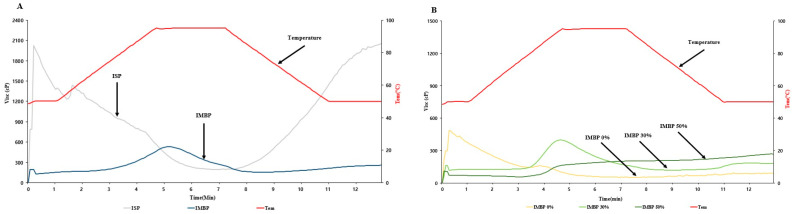
RVA viscosity curves of ISP and IMBP (**A**). RVA viscosity curves of IMBP 0%, IMBP 30%, and IMBP 50% (**B**). Isolated soy protein (ISP); isolated mung bean protein (IMBP).

The Rapid Visco Analyzer (RVA) is utilized for predicting viscosity changes in protein before extrusion [30]. Additionally, viscosity is an important parameter to consider during extrusion processes as it affects the flow behavior and mechanical energy input [31]. The viscosity of the raw materials of isolated soy protein (ISP) and isolated mung bean protein (IMBP), as well as mixtures with 0% (no added mung bean protein), 30%, and 50% added IMBP, is illustrated in Figure 2. Panel A of Figure 2 shows the viscosity of the raw materials. ISP initially exhibits a high initial viscosity of approximately 2000 cP, which then decreases as the powder absorbs water during hydration and subsequently decreases with increased mechanical and thermal energy. In contrast, the initial viscosity of IMBP is lower, at less than 200 cP, and rapidly increases upon heating to approximately 95 °C. Therefore, the lower initial viscosity of IMBP compared to ISP may be attributed to its higher solubility [32]. According to Benjakul, and Kishimura [33], IMBP has the highest hydrophobic amino acid content compared to isolated black bean protein and isolated peanut protein, reaching 53.1%. Additionally, hydrophobic amino acids play a crucial role in the thermal stability of globulins [34]. Thus, the sudden increase in the viscosity of IMBP at high temperatures can be attributed to its higher thermal stability. Panel B of Figure 2 displays the viscosity of mixtures containing IMBP. Compared to IMBP 30% and IMBP 50%, IMBP 0% reaches the shortest peak viscosity peak time at 1.07 ± 0.00 min. However, IMBP 50% has a peak time of 6.98 ± 0.04 min, requiring a longer time. This indicates that the addition of IMBP binds moisture and results in protein denaturation, thus requiring more time and higher temperatures compared to samples without added IMBP [35].

### 3.2. Specific Mechanical Energy

During the extrusion process, the specific mechanical energy (SME) input varies with the processing variables, such as moisture content, barrel temperature, screw speed, and feed rate. Therefore, the SME input is a key process parameter affecting the final extrudate’s appearance and physicochemical properties [36]. Table 1 illustrates the SME input as it varies with the addition of IMBP. Table 2 shows the F-values and *p*-values of the physicochemical and textural properties of high-moisture meat analogs with different isolated mung bean protein contents. When ISP content is replaced by 50% IMBP, the SME input reaches its highest value at 99.20 ± 0.19 kJ/kg. Brishti et al. [37] reported that, under the same experimental conditions, the minimum gelation concentrations for IMBP and ISP are 12% and 14%, respectively. A higher minimum gelation concentration indicates the lower gelation properties of the raw materials. Based on these results, it can be inferred that IMBP exhibits a relatively higher gelation ability compared to ISP, and as the content of IMBP increases, the viscosity of the mixture inside the barrel also increases. Additionally, the gelation ability of proteins indicates the degree of interaction between protein molecules, which correlates positively with viscosity [38]. The stronger the gelation ability of proteins, the higher the viscosity of the mixture after mixing with water in the barrel and the longer the residence time in the barrel, leading to a higher SME input required during the extrusion process. Therefore, it can be concluded that increasing the addition of IMBP leads to higher specific mechanical energy input due to enhanced gelation ability [39].

### 3.3. Appearance

**Figure 3:**
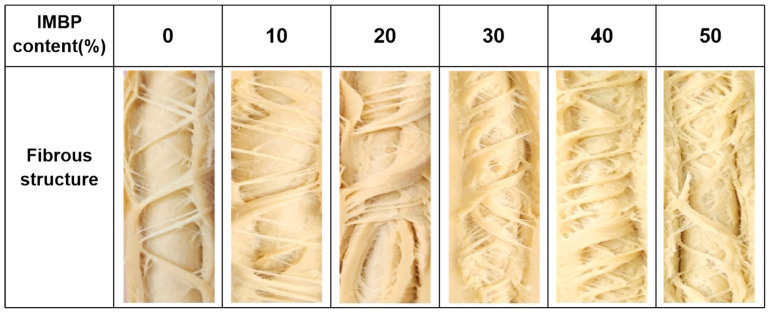
Photographs of the fibrous structure of the high-moisture extrusion meat analogs at different isolated mung bean different contents. Isolated mung bean protein (IMBP).

Figure 3 illustrates the appearance of the HMMAs produced based on varying levels of IMBP content. It can be observed that the content of IMBP is positively correlated with the fiber content of the HMMAs, with the highest fiber content achieved when the IMBP content reaches 40%. During the extrusion process, plant-based proteins exist as globular proteins before mixing with water. Upon hydration in the mixing zone, the size of globular protein particles increases, and a dispersed morphology is formed due to the encapsulation of free water by protein networks expanded by heating and shear forces [40]. It has been reported that the formation of fibrous structures in high-moisture meat analogs depends on the relative dynamics of protein aggregation and phase separation [41]. In the extrusion process with cooling dies, when the rate of protein aggregation is lower than the rate of phase separation, a stratified structure is observed [42]. Conversely, when the rate of protein aggregation exceeds the rate of phase separation, a non-oriented gel structure is formed [43]. According to Brishti et al. [37], compared to isolated soy protein, the isolated mung bean protein exhibits superior gelation ability. Increasing the content of isolated mung bean protein increases the viscosity of the protein melt inside the barrel, reducing the flowability of protein chains along the screw direction [44]. Therefore, the rate of protein aggregation through cooling dies is nearly equal to the rate of phase separation, thereby enhancing the formation of fibrous structures [13].

### 3.4. Water Holding Capacity

Water holding capacity (WHC) refers to the amount of water that can be bound to the structure of meat analogs during hydration processes [9]. Wi et al. [45] reported that a higher water holding capacity of meat analogs results in juicier products. Table 1 presents the water holding capacity of high-moisture meat analogs calculated based on the isolated mung bean protein content. The highest water holding capacity was observed when the IMBP content was 0%, reaching 4.39 ± 0.12 g/g, while the lowest was observed when the IMBP replaced 50% of ISP, at 2.12 ± 0.07 g/g. It is inferred that the increase in the viscosity of the mixture inside the barrel after adding the isolated mung bean protein reduces the space for water permeation through capillaries, thus decreasing the water holding capacity. Additionally, the water holding capacity of meat analogs is positively correlated with the resilience and viscosity, consistent with the findings of Samard and Ryu [26].

### 3.5. Water Absorption Capacity and Oil Absorption Capacity

**Figure 4:**
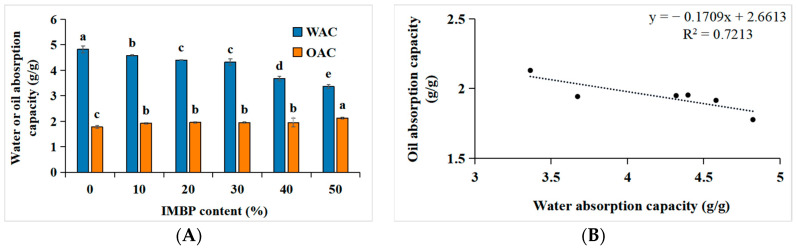
Water and oil absorption capacity of high-moisture extruded meat analogs according to the isolated mung bean protein (IMBP) content (**A**). Relationship between the oil absorption capacity (OAC) and water absorption capacity (WAC) of high-moisture extruded meat analogs according to the isolated mung bean protein content (**B**). Values are means of triplicates ± standard deviation. Values with different letters (^a–d^) in the same bar indicate significant differences (*p* < 0.05) by Duncan’s multiple range test.

The water absorption capacity (WAC) and oil absorption capacity (OAC) of proteins are crucial for the product quality, including juiciness, texture, mouthfeel, reduction in moisture loss, preservation, and flavor retention [46]. Figure 4A,B depict the correlation between the WAC and OAC of high-moisture meat analogs and the content of IMBP. When IMBP is at 0%, the WAC is highest, at 4.82 ± 0.13 g/g, gradually decreasing with the increase in the content of isolated mung bean protein. Conversely, when IMBP reaches 50%, the OAC is highest, at 2.13 ± 0.04 g/g, gradually increasing with the content of IMBP. The decrease in WAC may be attributed to shear forces and pressures during the extrusion process leading to protein structural denaturation [9], exposing hydrophobic amino acids originally located internally [47]. Consequently, hydrogen bonding between hydrophilic amino acids and water weakens [48]. However, with the increase in the IMBP content, the OAC also increases. This is because the exposed hydrophobic amino acids in denatured proteins increase, allowing them to bind with oil [49]. Furthermore, the correlation between the WAC and OAC with the content of IMBP is illustrated in Figure 4B. As the WAC increases, the OAC decreases, showing a negative correlation (R_2_ = 0.7213). In summary, as the OAC increases with the increase in IMBP content, meat analogs containing IMBP are more suitable as intermediates compared to those containing ISP, particularly for flavor enhancement and texture improvement [50].

### 3.6. Integrity Index and Nitrogen Solubility Index

**Figure 5:**
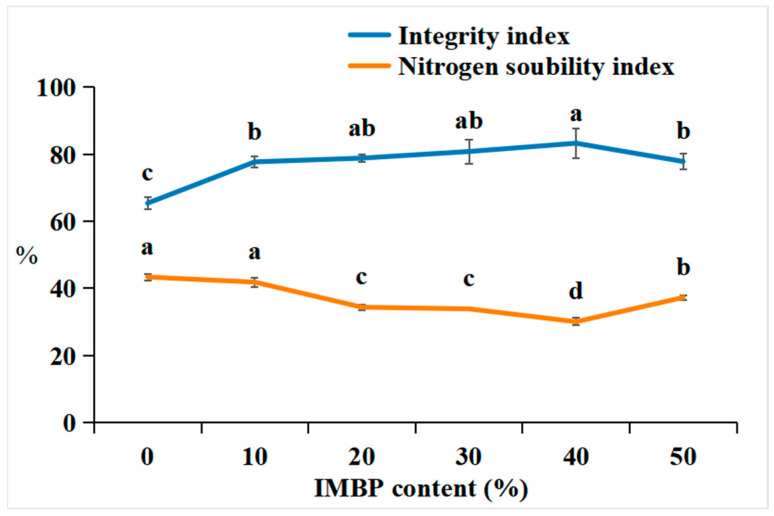
Correlation between the integrity index and nitrogen solubility index of high-moisture extruded meat analogs according to the isolated mung bean protein (IMBP) content. Values are means of triplicates ± standard deviation. Values with different letters (^a–d^) in the same lineindicate significant differences (*p* < 0.05) by Duncan’s multiple range test.

The integrity index is determined by measuring the residual residue of meat analogs after the hydration, pressing, homogenization, and drying processes, which ultimately affects the quality and yield of the meat analogs [51]. Figure 5 shows the integrity index and nitrogen solubility index (NSI) of the HMMAs with different contents of IMBP. The integrity index of the HMMAs increases when the content of IMBP reaches 40% and decreases when it reaches 50%. In texture analysis, the addition of IMBP increased the texture factor (chewiness) by 40%, attributed to the formation of a strong gel network between protein molecules, which can maintain and preserve the simulated meat structure even under high-temperature and high-pressure conditions. A lower integrity index was observed in samples with 50% IMBP content compared to those with 40%, possibly due to a reduction in disulfide bonds.

The NSI is an important indicator for assessing the degree of protein denaturation during tissue processing [52]. The NSI of the HMMAs decreases until the content of IMBP reaches 40% and then increases again at 50%. The decrease in NSI at 40% is attributed to protein denaturation during high-moisture extrusion. This denaturation is believed to increase protein–protein interactions [51], and IMBP forms relatively stronger gels in the subsequent 50%. This results in a decrease in shear forces acting on the protein gel network dispersed in the barrel due to reduced intra- and intermolecular interactions. Therefore, the binding strength of the new protein network decreases [53].

### 3.7. Texture Profile Analysis and Cutting Strength

The texture of meat analogs is a critical factor in mimicking the sensory taste of real meat [45]. Resilience measures the extent to which a sample recovers after deformation caused by external forces, while cohesion represents the strength of internal binding [54]. Chewiness is considered the most crucial factor in expressing texture sensory impressions [41]. The texture and cutting strength of high-moisture meat analogs are shown in Table 3. When the content of IMBP is 0%, the resilience is the highest, at 91.26 ± 0.82%. However, the resilience decreases with the increase in the IMBP content. As the IMBP content increases, the protein content of meat analogs decreases, resulting in decreased resilience. This is consistent with the findings of Jeon, Gu, and Ryu [55], which showed that the resilience of high-moisture meat analogs decreases with the increase in the yeast content. Moreover, when the IMBP content is 0%, cohesion is the highest, at 76.89 ± 2.45%. However, cohesion decreases with the increase in the IMBP content to 50%, reaching 55.81 ± 1.77%. This is because protein–protein interactions between ISP molecules are stronger than those between IMBP molecules, leading to a decrease in the internal binding strength with the increase in the IMBP content [39]. There were no significant differences in chewiness between 10% and 40% (including ISP and IMBP). These results may be attributed to the enhanced interactions between ISP and IMBP, forming a strong gel network [33]. On the other hand, the chewiness of 50% IMBP is the lowest, at 3649.69 ± 281.40 g. This is due to a reduction in disulfide bonds, which are key components in forming the three-dimensional internal structure of meat analogs [56]. A study comparing the sulfur amino acid content of ISP and IMBP also supports these results, indicating a relatively lower sulfur amino acid content in IMBP compared to ISP [57].

The cutting strength of meat analogs determined by the IMBP content indicates that all samples have a higher cutting strength in the vertical direction than in the flow direction. These results suggest that all samples exhibit a fibrous structure, consistent with the report by Webb, Li, and Alavi [58]. Compared to samples containing both ISP and IMBP, samples with 50% IMBP replacing ISP have lower values in resilience, cohesion, chewiness, and cutting strength. These results are aligned with the findings of Samard and Ryu [59], who reported that overall texture characteristics of meat analogs based on IMBP are lower compared to those based on ISP. In conclusion, the complete replacement of ISP with IMBP may lead to a decrease in the overall texture characteristics. However, it holds promise as an intermediate material helpful in adjusting texture characteristics [58].

## 4. Conclusions

The addition of isolated mung bean protein leads to changes in the fibrous structure of high-moisture meat analogs. When the content of isolated mung bean protein is 40–50%, the fibrous structure closely resembles that of real meat. In the texture profile analysis (TPA), the chewiness of the HMMAs is relatively high when the content of isolated mung bean protein is in the range of 10–40%. This suggests its potential direct use in products. However, when isolated mung bean protein replaces 50% of isolated soy protein, the resulting HMMA texture is softer with a lower chewiness. Therefore, it is recommended as an intermediate material to be mixed with various ingredients. In plant proteins, oil plays a crucial role in achieving texture like that of animal proteins. The highest oil absorption capacity is observed when the content of isolated mung bean protein is 50%, indicating that isolated mung bean protein is more suitable than isolated soy protein for making plant-based meat analogs and dairy products. Further research is needed to optimize the variables by changing independent variables, such as the moisture content, screw speed, and barrel temperature, to find suitable meat analogs for processing. This study demonstrates the potential and feasibility of using isolated mung bean protein instead of isolated soy protein, which is currently the main ingredient used in meat analog production.

## Figures and Tables

**Table 1:** Specific mechanical energy input and water holding capacity of the high-moisture extrusion meat analog according to the content of isolated mung bean protein.

IMBP Content (%)	Specific Mechanical Energy		Water Holding Capacity	
	Mean ± SD (%)	Standard Error	Mean ± SD (%)	Standard Error
0	46.26 ± 0.34 ^f^	0.20	4.38 ± 0.17 ^a^	0.10
10	70.02 ± 0.46 ^e^	0.27	3.49 ± 0.13 ^b^	0.08
20	87.20 ± 0.52 ^d^	0.30	3.16 ± 0.59 ^b^	0.34
30	91.74 ± 1.16 ^c^	0.67	2.54 ± 0.17 ^c^	0.10
40	96.67 ± 0.61 ^b^	0.35	2.23 ± 0.03 ^c^	0.02
50	99.19 ± 0.19 ^a^	0.11	2.12 ± 0.07 ^c^	0.04

Values with different letters (^a–f^) in the same column indicate significant differences (*p* < 0.05) by Duncan’s multiple range test. IMBP: Isolated mung bean protein.

**Table 2:** The F-values and *p*-values of the physicochemical and textural properties of the high-moisture meat analogs with different isolated mung bean protein contents.

	SME	WHC	WAC	OAC	II	NSI
F value	3135.500	31.977	114.762	6.493	14.895	88.625
*p* value	<0.000	<0.000	<0.000	0.004	<0.000	<0.000

SME: specific mechanical energy, WHC: water holding capacity, WAC: water absorption capacity, OAC: oil absorption capacity, II: integrity index, NSI: nitrogen solubility index.

**Table 3:** Texture properties of high-moisture extrusion meat analogs according to the IMBP content.

IMBP Content (%)	Springiness (%)	Cohesiveness (%)	Chewiness (g)	Cutting Strength (g/cm^2^)	
				Vertical Direction	Parallel Direction
0	91.26 ± 0.82 ^a^	76.89 ± 2.45 ^a^	4724.33 ± 455.26 ^b^	915.68 ± 119.57 ^d^	905.28 ± 53.85 ^a^
10	91.60 ± 0.75 ^a^	76.14 ± 1.03 ^ab^	5817.87 ± 489.18 ^a^	1158.92 ± 126.64 ^b^	901.25 ± 83.73 ^a^
20	91.68 ± 0.70 ^a^	73.27 ± 3.41 ^bc^	5436.76 ± 190.11 ^a^	1085.63 ± 43.65 ^bc^	894.49 ± 53.68 ^a^
30	91.15 ± 0.49 ^a^	73.16 ± 2.27 ^bc^	5591.23 ± 372.06 ^a^	1321.03 ± 193.65 ^a^	732.53 ± 48.51 ^b^
40	90.15 ± 1.38 ^b^	70.17 ± 4.42 ^c^	5441.18 ± 492.71 ^a^	959.85 ± 26.26 ^cd^	721.16 ± 53.03 ^b^
50	84.49 ± 0.58 ^c^	55.81 ± 1.77 ^d^	3649.69 ± 281.40 ^c^	746.18 ± 49.58 ^e^	632.89 ± 23.12 ^c^

IMBP: Isolated mung bean protein. Values with different letters (^a–e^) in the same column indicate significant differences (*p* < 0.05) by Duncan’s multiple range test.

## Data Availability

The original contributions presented in the study are included in the article, further inquiries can be directed to the corresponding author.
